# Intraoral Lipoma: A Case Report

**DOI:** 10.1155/2014/480130

**Published:** 2014-01-30

**Authors:** L. K. Surej Kumar, Nikhil Mathew Kurien, Varun B. Raghavan, P. Varun Menon, Sherin A. Khalam

**Affiliations:** Department of Oral & Maxillofacial Surgery, PMS College of Dental Science & Research, Golden Hills, Vattappara, Venkode, Thiruvananthapuram 695028, India

## Abstract

Lipomas are rare in oral and maxillofacial regions although they are the most common tumours of mesenchymal origin in human body. The etiology remains unclear. Various different theories explain the pathogenesis of this adipose tissue tumour and also different histological variants of oral lipoma have been given in literature. A case of intraoral lipoma occurring in mental region in a 77-year-old male is reported along with review of the literature. Wide surgical excision was performed and two-year followup showed excellent healing without any recurrence. Lipomas are benign soft tissue neoplasm of mature adipose tissue seen as a common entity in the head and neck region. Intraoral lipomas are a rare entity which may be noticed only during routine dental examinations. Most of them rarely cause pain, resulting in delay to seek treatment. It is mandatory for a clinician to diagnose intraoral lipomas using latest diagnostic methods and conservatively treat them without causing much discomfort.

## 1. Introduction

Lipomas are the most common soft tissue neoplasm, representing 0.1 to 5% of all benign tumors of the mouth. About 15 to 20% of the cases involve the head and neck region, while 1–4% affect the oral cavity, an uncommon site for the occurrence of lipoma [1, 2]. Usually they are seen as long-standing soft nodular asymptomatic swellings covered by normal mucosa. They particularly occur in the areas of fat accumulation, especially the cheek, followed by the tongue, floor of the mouth, buccal sulcus and vestibule, lip, palate, and gingiva [3, 4]. Histologically, they can be classified as simple lipoma, fibrolipoma, spindle cell lipoma, intramuscular or infiltrating lipoma, angiolipoma, pleomorphic lipoma, myxoid lipoma, and atypical lipoma. Intramuscular or infiltrating lipoma is an uncommon mesenchymal tumor, usually appearing in the extremities or trunk but rarely occurring in the oral cavity [5]. Oral infiltrating lipomas are larger than the ordinary oral lipomas and present clinically as deep-seated, slow growing, painless masses [6]. Here we report a case of intraoral lipoma along with the review of literature.

## 2. Presentation of Case

**Figure 1:**
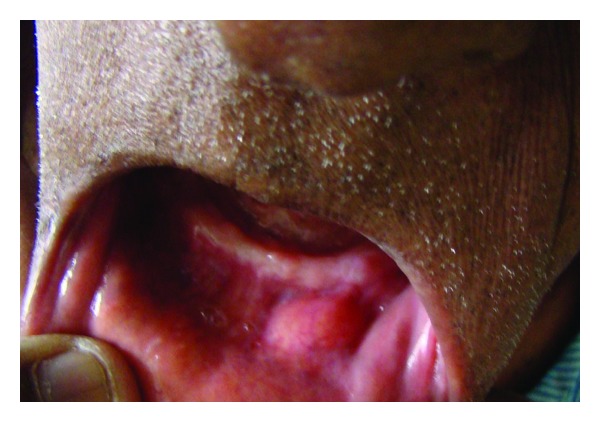
Intraoral swelling in relation to 34, 35 region.

A 77-year-old male patient was referred to the Department of Oral and Maxillofacial Surgery with the complaint of swelling in relation to lower left mental region for the past year. The patient also complained of discomfort and feeling of heaviness in the area of the swelling. His medical history was noncontributory. Extra oral examination revealed a diffuse swelling in the left lower mental region, measuring 2 × 1 cm. On palpation, the nature of the swelling was mobile, firm, and nontender. Intraorally it presented as a yellowish, oval swelling in the buccal left sulcus in relation to 34, 35 region (Figure 1). The covering mucosa was normal in texture without ulceration or inflammation.

**Figure 2:**
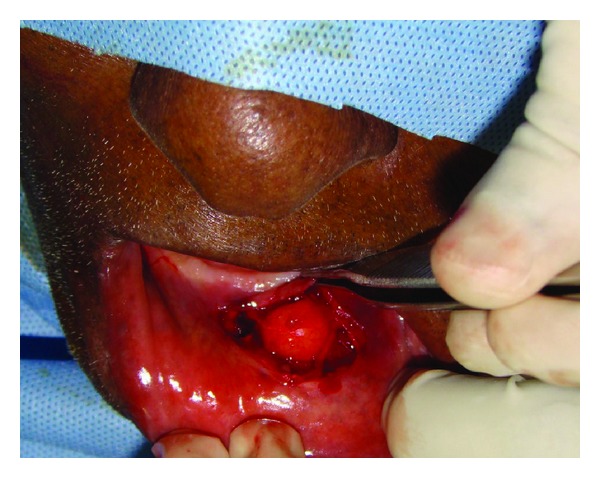
Exposed irregular, poorly encapsulated, and lobulated mass.

**Figure 3:**
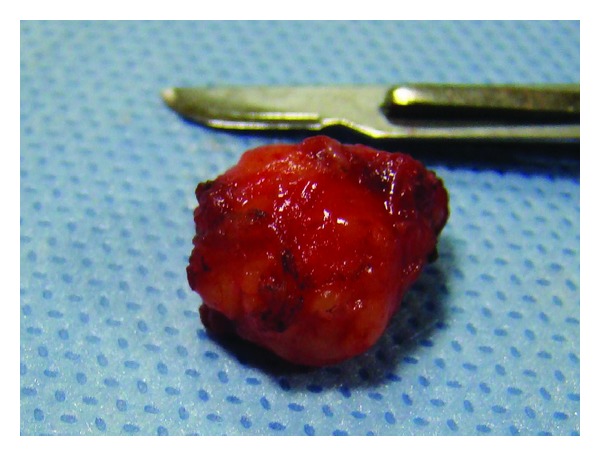
Excised specimen.

Excision biopsy was planned under local anesthesia. Blunt dissection was performed; the mucous membrane was undermined exposing an irregular, poorly encapsulated, and lobulated pale yellow mass (Figure 2). Excised specimen was 2 × 1 cm in size (Figure 3) and was sent for histopathological examination. A review after 7 days showed uneventful healing and the sutures were therefore removed. Two-year followup revealed no recurrence.

**Figure 4:**
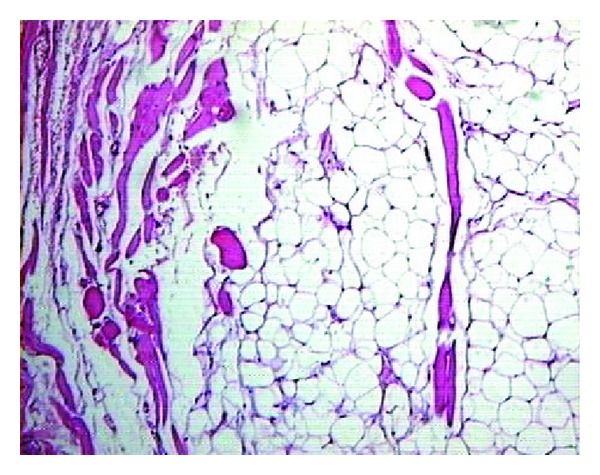
Microscopic view showing the characteristic features.

The histopathology of the soft tissue section showed an encapsulated lesion composed of abundant mature adipocytes arranged in lobules. The lobules are separated by fibrous connective tissue septa. The adipocytes appeared polygonal in shape with clear cytoplasm and eccentrically placed nucleus that was compressed against the cell membrane. Dilated blood capillaries, extravasated RBCs, muscle fibers, and minimal inflammatory cells were seen in the deeper part of the section. Lobules of mucous salivary acini were also seen associated with the lesional tissue (Figure 4).

## 3. Discussion

Lipomas are benign soft tissue neoplasm of mature adipose tissue seen as a common entity in the head and neck region. Intraoral lipomas are rare, the statistics showing only 1 to 4% affecting these sites [1, 2]. Furlong et al. found only 125 cases of oral lipomas over a period of 20 years, which again shows the rarity of this oral tumours [7]. The first description of an oral lesion was provided by Roux in 1848, in a review of alveolar masses which he referred to as “yellow epulis” [8].

The etiology of intraoral lipoma remains unclear, but the suggested pathogenic mechanisms include the “hypertrophy theory” which states that obesity and inadvertent growth of adipose tissue may contribute to formation of these oral lesions. This theory is less convincing in explaining those lesions occurring in areas devoid of preexisting adipose tissue [9]. They are not used up in general metabolism during periods of starvation like normal adipose tissue.

Another theory known as “metaplasia theory” suggests that lipomatous development occurs due to aberrant differentiation of *in situ* mesenchymal cells into lipoblast, since fatty tissue can be derived from mutable connective tissue cells almost anywhere in the body [10]. J. J. Lin and F. Lin suggested that these benign entities are congenital lesions arising from embryonic multipotential cells that remain subclinically dormant until they differentiate into fat cells under hormonal influence during adolescence [11]. However, in some cases, trauma and chronic irritation may trigger the proliferation of soft tissue and play a role in the development of a lipoma [3].

A review of 26 cases done by de Freitas et al. in the Brazilian population showed that the mean age of occurrence is 54.6 years [12]. Fornage and Tassin reported that the peak incidence occurs in the fifth or sixth decade of life [13]; whereas, rare cases of congenital lipomas have been reported in 20-day and 47-day old babies [14]. This benign tumour occurs predominantly in females [1], while literature showing equal sex distribution with a male-female ratio of 1 : 1.2 has also been reported [14].

Lipomas have been reported in all parts of the body including regions of back, shoulder, neck, and extremities [12]. Intraoral counterparts are rare; most common site of oral lipomas is the oral mucosa, a region rich in fatty tissue, followed by the tongue, lips, floor of the mouth, palate, and gingival. This pattern corresponds to the quantity of fat deposits in the oral cavity [4, 15]. Rare cases of intraosseous lipomas have been described by Oringer and Johnson in the body of mandible and ramus, respectively [16, 17].

The clinical features may vary according to the location of the lesion. Usually they manifest as slow growing, sessile round to avoid submucosal nodules. Unless the yellow colour of the tumour appears through the overlying thin mucosa, diagnosis of these tumours clinically is not always easy [18]. The size may vary from 0.2 to 1.5 cm in diameter, although tumours as large as 50 mm have been reported in the cheek [6]. Signs and symptoms may include a feeling of fullness and discomfort. Rarely various functional problems like dysphagia, difficulty in speech, and mastication have also been encountered in large sublingual lipomas. Literature review has shown that 5% of the cases were multiple. Multiple lipomas have been associated with certain syndromes like neurofibromatosis, Gardner's syndrome, painful multiple subcutaneous lipomas and obesity syndrome called Decrum's disease, encephalocraniocutaneous lipomatosis, multiple familial lipomatosis, Proteus syndrome, and Pai syndrome [19].

The differential diagnosis of intraoral lipoma includes oral dermoid and epidermoid cysts, oral lymphoepithelial cyst, benign salivary gland tumour, mucocele, benign mesenchymal neoplasm, ranula, ectopic thyroid tissue, and lymphoma. Lesions appearing as swelling on the dorsum of the tongue usually mimic hemangioma, lymphangioma, rhabdomyoma, neuroma, or neurofibroma.

The diagnosis of intraoral lipomas is usually clinical. Techniques like xeroradiography and echography are often used to delineate the anatomical extent of intraoral lesions but have limited capacity to precisely determine the extent of the lesion.

Computed tomography and magnetic resonance imaging enable the diagnosis of these tumours to be made quite readily. In spite of availability of all these techniques, histopathology remains the gold standard in the diagnosis of lipoma [6].

Histologically, the tumor is composed of adult fat cells that are subdivided into lobules by fibrous connective tissue septa. Based on microscopical features they are classified into classic lipoma, fibro lipoma, angiolipoma, spindle cell lipoma, and pleomorphic, myxoid, sialolipoma, and intramuscular lipomas [3]. Among these variants, myxoid lipomas and angiolipomas are rarely found in the oral cavity [14]. Diversity in histological pattern like dense fibrous connective tissue septa, spindle cell components, mitotically active atypical cells, mature blood vessels, myxoid stroma, or even salivary acinar structures is seen along with mature adipose tissue depending on each variant [3].

Intramuscular or infiltrating lipoma is an unusual clinical variant of this adipose tissue neoplasm, originating between skeletal muscle bundles and infiltrating through the intramuscular septa. They have a slight predilection for the tongue, due to the close relationship between the adipose tissue and the muscular layer. In infiltrating lipomas, there is a consistent and diffuse infiltration with dissociation and entrapment of the muscle fibers, some of which show degenerative changes. The muscle tissue is replaced by the fat, which may extend beyond the muscle fascia into the intermuscular connective tissue spaces. Fascia, joint capsules, bones, and nerves may also be infiltrated. Infiltrative lipomas could suggest a false diagnosis of liposarcoma but absence of cellular pleomorphism, nuclear hyperchromatism and low mitotic activity support the diagnosis of intramuscular lipoma [5].

Main stay of treatment for intraoral lipoma is complete surgical excision. No recurrence has been described after local excision, but infiltrative lipoma tends to recur after inadequate excision due to the fact that they are not encapsulated like simple lipomas. Even in cases with recurrence there has been no reported incidence of malignant transformation [5].

Medical management of lipomas, which is now common, includes steroid injections that result in local fat atrophy, thus, shrinking the tumour size. They are best done on lipomas that are less than 1 inch in diameter. A monthly repeated injection of 1 : 1 mixture of lidocaine and triamcinolone acetonide into the central region of tumour may be useful in regression of lesion.

Average volume of steroid used may range from 1 to 3 mL depending on the size of tumour. Liposuction using a 16-gauge needle and large syringe are useful in small or large lipomatous growth where scarring should be avoided.

## 4. Conclusion

Intraoral lipomas are a rare entity which may be noticed only during routine dental examinations. Most of them rarely cause pain, resulting in delay to seek treatment. The patient's concerns may be regarding esthetics or discomfort. It is mandatory for a clinician to diagnose intraoral lipomas using latest diagnostic methods and conservatively treat them without causing much discomfort. Newer nonsurgical treatment modalities are still under trial which may come into practice in recent future.
